# First experimental results of the cosmic time synchronizer for a wireless, precise, and perpetual time synchronization system

**DOI:** 10.1016/j.isci.2023.106595

**Published:** 2023-04-07

**Authors:** Hiroyuki K.M. Tanaka, Giancarlo Cerretto, Ivan Gnesi

**Affiliations:** 1 University of Tokyo, Tokyo, Japan; 2 International Virtual Muography Institute, Global, Tokyo, Japan; 3 Quantum Metrology and Nanotechnologies Division, Istituto Nazionale di Ricerca Metrologica, Torino, Italy; 4 Fermi Research Center (CREF), Rome, Italy; 5 European Center for Nuclear Research (CERN), Geneva, Switzerland; 6 Istituto Nazionale di Fisica Nucleare, Frascati National Labs - Cosenza Group - Cosenza, Frascati, Italy

**Keywords:** Particle astrophysics, High-energy astrophysics, Telecommunication engineering

## Abstract

In 2022, the idea of the cosmic time synchronizer (CTS) was proposed for a precise wireless synchronization of local clocks (<100 ns). Because CTS does not require critical timing information traffic among CTS sensors, the technique can be considered robust against jamming and spoofing. In this work, a small-scale CTS sensor network has been developed and tested for the first time. Good time synchronization performances were obtained for a short-haul configuration (30–35 ns (SD, 1 σ), over 50–60 m). Based on the results of this work, CTS could be potentially conceived as a ‘self-adjusting’ system, offering high level continuous (perpetual) performances, to be considered either as a backup chain for GPS disciplined oscillators (GPS DO), a standalone standard for frequency and time interval measurements, or as a tool for the dissemination of reference time scales to final users, with improved characteristics in terms of robustness and reliability.

## Highlights

• CTS offers secure, robust, perpetual time synchronization without drift
• GPS backup performance of 35 ns over a 60-m nodal distance
• CTS sensor network has been developed and demonstrated for the first time

## Graphical abstract

**Figure:**
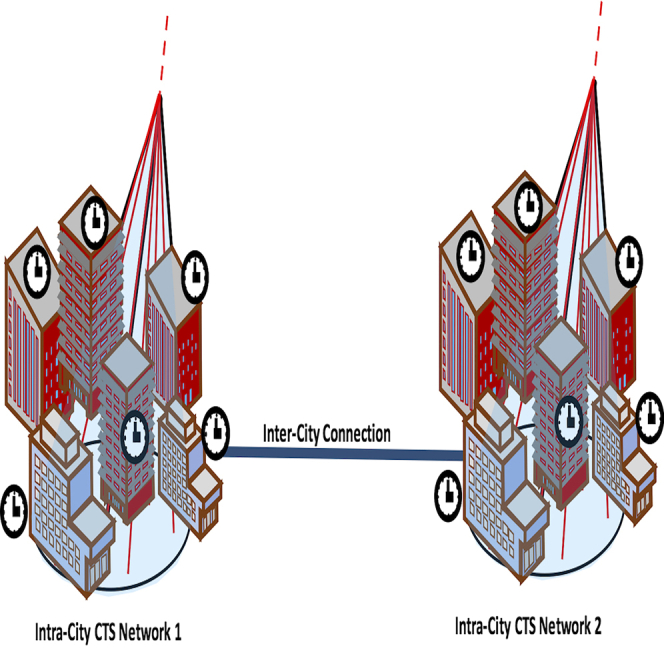

## Introduction

Global positioning system (GPS) and other GNSS (global navigation satellite systems; hereafter GPS) are currently employed for a large variety of applications, from positioning (mass-market and high precision) to providing access to reference time scales, either the (atomic) system timescale (i.e., GPS Time), the Coordinated Universal Time (UTC) computed by BIPM (International Office for Weight and Measurements) or a UTC physical realization, UTC(k), generated at National Metrology Institutes (NMIs) or Astronomical Observatories. The possibility to access to a reference (atomic) timescale is an important added value for GPS, making it a standard and a widely used system for the remote comparison of atomic clocks and time scales, as well as for the dissemination of time reference to final users.

Accurate, stable, secure, and reliable time references are in fact an important prerequisite for a wide range of applications, including financial transactions,^1^ operation and regulation of factory robots,^2^ transfer and generation of electric power,^3^ homeland security,^4^ scientific experiments,^5^ as well as Time Metrology Applications.^6^ Making use of stand-alone, highly accurate and stable local atomic clocks is a viable solution to provide required time synchronization, both in terms of metrological characteristics (i.e., accuracy and precision/stability) and robustness/reliability. For example, commercial high performance Cesium beam clocks, could reach frequency accuracy at the level of 5 × 10^−13^, corresponding to a time rate of about 40 ns/day, which allows for moderately appropriate time synchronization, over relatively extended periods, depending on the considered applications. However, because of the high cost of commercial Cesium beam clocks this is not always a practical or convenient solution. Another commonly used option for high precision time synchronization, especially for industries and productive sectors, is the adoption of the GPS Disciplined Oscillators, which are less costly oscillators (typically OCXO or Rubidium), equipped with an embedded GPS reception module and related antenna, allowing the oscillator to be frequency locked to the GPS Timescale. GPS DOs – in fact - combine the short-term stability of the local clock, with the long-term one, provided by the GPS Time atomic timescale. Obviously, excellent, and consistent GPS signal availability is mandatory for systems like this, which anyhow could have specific drawbacks coming – for example – from potential jamming and spoofing attacks.

GPS, in fact, having low-power and unencrypted signals, is vulnerable to both intentional and unintentional disruption. The US Department of Homeland Security has launched a positioning, navigation, and timing (PNT) Program to address this issue. They write in their website:

“To address GPS vulnerabilities in critical infrastructure, the Science and Technology Directorate (S&T) Positioning, Navigation, and Timing (PNT) Program has a multi-pronged approach of conducting vulnerability and impact assessments, developing mitigations, exploring complementary timing technologies, and engaging with industry through outreach events and meetings. Through these sustained efforts, the goal of the program is to increase the resiliency of critical infrastructure to GPS vulnerabilities in the future (US Department of Homeland Security, 2022).”

Because of the inherent characteristics of GPS mentioned above, strategies for dealing with jamming and spoofing risks are crucial to grant for timing robustness and resilience in critical infrastructures. Different GPS anti-spoofing (detection and mitigation) techniques have been proposed, including amplitude discrimination, time of arrival (TOA) discrimination, consistency cross-check with inertial measurement units (IMU), polarization discrimination, angle of arrival (AOA) discrimination and cryptographic authentication.^7^^,^^8^^,^^9^^,^^10^ However, most of these techniques focus on spoofing detection, rather than spoofing mitigation. One of the established techniques for the mitigation of GPS spoofing makes use of multiple antennas.^7^^,^^11^^,^^12^^,^^13^^,^^14^ For example, the technique that has been proposed by McDowell (2007)^13^ utilizes an antenna array to compare AOAs after they are fully tracked by the GPS receiver; however, this method requires a high computational load on the receiver.

When applicable, wired systems could grant for more secured synchronization, with respect to GPS. For example, high security is required for electronic power conditioning, control of production, and distribution of electricity (i.e., power grids), leading electric companies to install their own dedicated wired time synchronization systems.^15^ These wired systems can be based on quartz or silica optical fibers with reduced low temperature sensitivity (100 ps K^−1^ km^−1^), which improve the time stability in the remote clocks synchronization.^16^ In addition, if the clocks synchronization is based on informatics protocols over wired networks, security is further strengthened. However, because the actual time information is physically transferred through the network, a residual hacking/spoofing risk for the system is still present.

Although highly secure, accurate and stable time synchronization techniques are becoming an important prerequisite for an increasing number of applications, it is rather difficult to find a perfect and transversal trade-off between performances, robustness, and reliability.

In this work, we present the world’s first successful experimental results of a completely new and robust time synchronization technique, called Cosmic Time Synchronization (CTS), making use of extensive air shower (EAS) particles. CTS is a technique invented by Tanaka (2022)^17^ as an innovative, accurate, precise, and secure time synchronization system, making use of the simultaneity characteristics of EAS particles. In the following section, more details about the CTS principle of functioning will be described. Then, the results of a preliminary experimental evaluation of the CTS time synchronization capabilities will be proposed, based on a small-scale CTS daisy chain (∼100 m) network, implemented at Tokyo University Laboratories.

## Results

### Extensive air showers (EAS) and CTS

**Figure 1:**
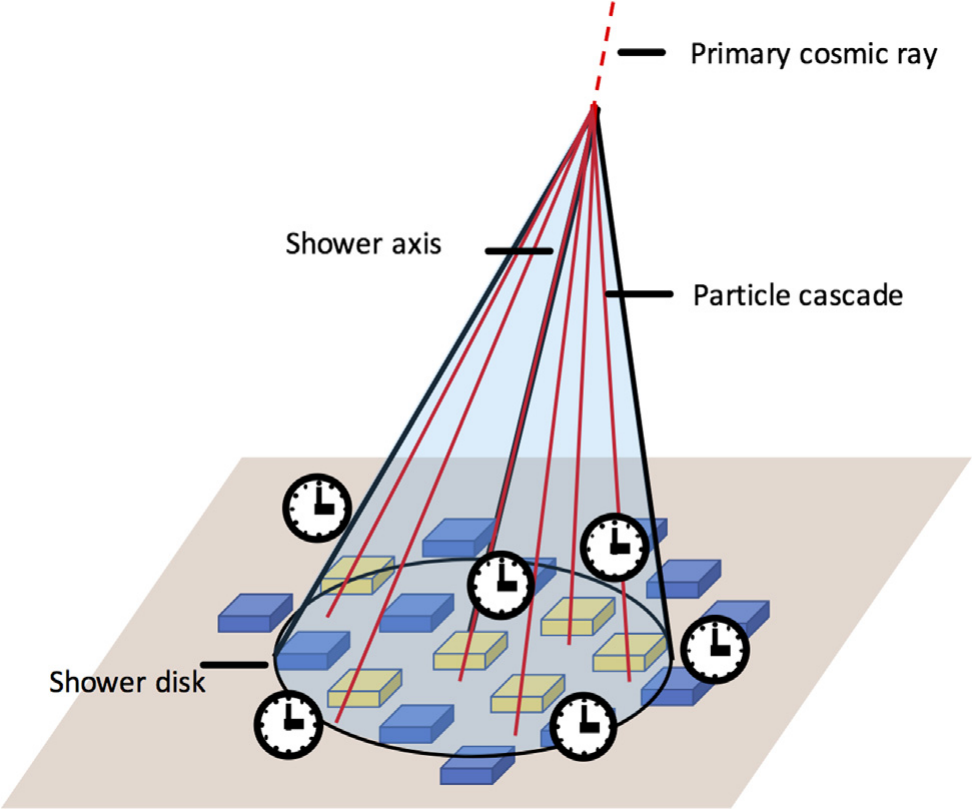
CTS concept Blue and yellow boxes indicate two-dimensionally deployed CTS sensors. Yellow boxes are CTS sensors within the EAS coverage area.

CTS takes advantage of the simultaneity characteristics of remote EAS particles, which are generated in the Earth’s atmosphere and travel to the ground surface (and sometimes beyond). As indicated in Figure 1, in the high-energy interactions of the primary cosmic rays with atmospheric nuclei, cascades of secondaries (electromagnetic cascade and hadronic cascade) are generated. The resulting EAS spread out over large areas. The lateral distribution function (LDF) of EAS particles can be approximated by Greisen’s function^18^^,^^19^ as a function of lateral distance, where the lateral distance is defined as the distance from the shower axis.

(Equation 1) $$\rho_{\mu }\left(r\right)=\rho_{\mu }\left(r_{0}\right){\left(\frac{r}{r_{0}}\right)}^{-3/4}{\left(\frac{320+r}{320+r_{0}}\right)}^{-r}$$

This particle lateral distribution function is used by all EAS experiments to measure for each event, the coordinates of the core, the slope of the function and the normalization parameter that are then used to determine the energy of the primary particle. Several experiments have attempted to fit the parameters gamma and ρμ(*r*_0_).^20^^,^^21^^,^^22^ The choice of *r*_0_ is arbitrary, depending on what spatial-scale of EASs are discussed. For example, for reconstructing muon lateral distribution with IceCube, 600 m was chosen as the value for *r*_0_.^19^

The electromagnetic showers contain only electrons, positrons and gamma particles, produced by Bremsstrahlung and pair-production processes. The electromagnetic component of the hadronic air shower is generated principally by neutral pion decaying into two photons and initiating electromagnetic sub-showers. On the other hand, muons are generated from high energy mesons in the hadronic cascade (charged pions and kaons). A large fraction of these muons are relativistic in nature and they penetrate the atmosphere losing only a small fraction of their energy predominantly by ionization. The penetrative nature of muons has been utilized to visualize the inside structures of gigantic objects such as volcanoes,^23^^,^^24^^,^^25^^,^^26^^,^^27^^,^^28^^,^^29^^,^^30^^,^^31^^,^^32^^,^^33^^,^^34^^,^^35^^,^^36^^,^^37^^,^^38^^,^^39^ local ocean dynamics^40^ cultural heritage,^41^^,^^42^^,^^43^^,^^44^^,^^45^^,^^46^ underwater/underground positioning^47^ and navigation,^48^ and postquantum cryptography.^49^

The lateral spread of the muon component is mainly caused by the transverse momenta of the parent mesons; furthermore, compared to the electron, the muon’s multiple scattering contribution is much smaller because it is suppressed by a factor of (*m*_e_/*m*μ).^2^ Shower particles close to the shower axis will travel the shortest path from the point of generation to the ground surface, but as the distance from the shower axis increases, the path from the point of generation to the ground surface of these corresponding particles will increase. The path length difference and variations in the velocities of the secondaries contribute to the longitudinal spread (the shower disk thickness). Also, the difference between paths traveled by different particles on the same EAS comes from the direction of the EAS axis. If the axis is strongly inclined, some EAS particles will reach the ground surface before other EAS particles; detectors which are situated closer to the axis will receive the particles before detectors, which happen to be further from the axis. Electromagnetic particles are generally produced deeper in the atmosphere and closer to the shower axis in comparison to muons. When detected at the ground level, these electromagnetic particles tend to originate from deeper in the atmosphere or at a later stage of the shower development. Therefore, electromagnetic particles have lower average kinetic energy compared to muons. The CTS technique can make use of all the detected charged particles for time synchronization. However, in underground/underwater environments deeper than 10 radiation lengths (5.9 m.w.e. in SiO_2_/3.6 m in water), because electromagnetic flux is significantly reduced, the considered particles are mainly muons. Because the minimum energies of muons that can penetrate these thicknesses of SiO_2_ and water are respectively 1.4 GeV and 0.8 GeV, it is anticipated that CTS works at least up to these depths. CTS is based on arrivals of EAS particles, timing information traffic is not needed among CTS sensors. In this sense, although the timing information seems to be distributed to CTS sensors as if it is teleported, additional data transfer is required for verification of the timing information.

### EAS Monte Carlo simulations

In the current work, because the positions of the shower axes are not the focus of interest, the only requirement was to estimate the rate of coincidence as a function of distance between the ground detectors. EAS simulations were performed by using the AIRES Monte Carlo EAS simulation package^50^ to estimate the number of EAS particles available for the current CTS experiment and the attainable synchronization accuracy. SIBYLL 2.3days^51^ was used as the hadronic interaction model, and the extended Linsley’s standard atmosphere model^52^ was employed in the current simulations. For calculations of magnetic deflections, a model based on experimental data called the international geomagnetic reference field (IGRF)^53^ was used.

**Figure 2:**
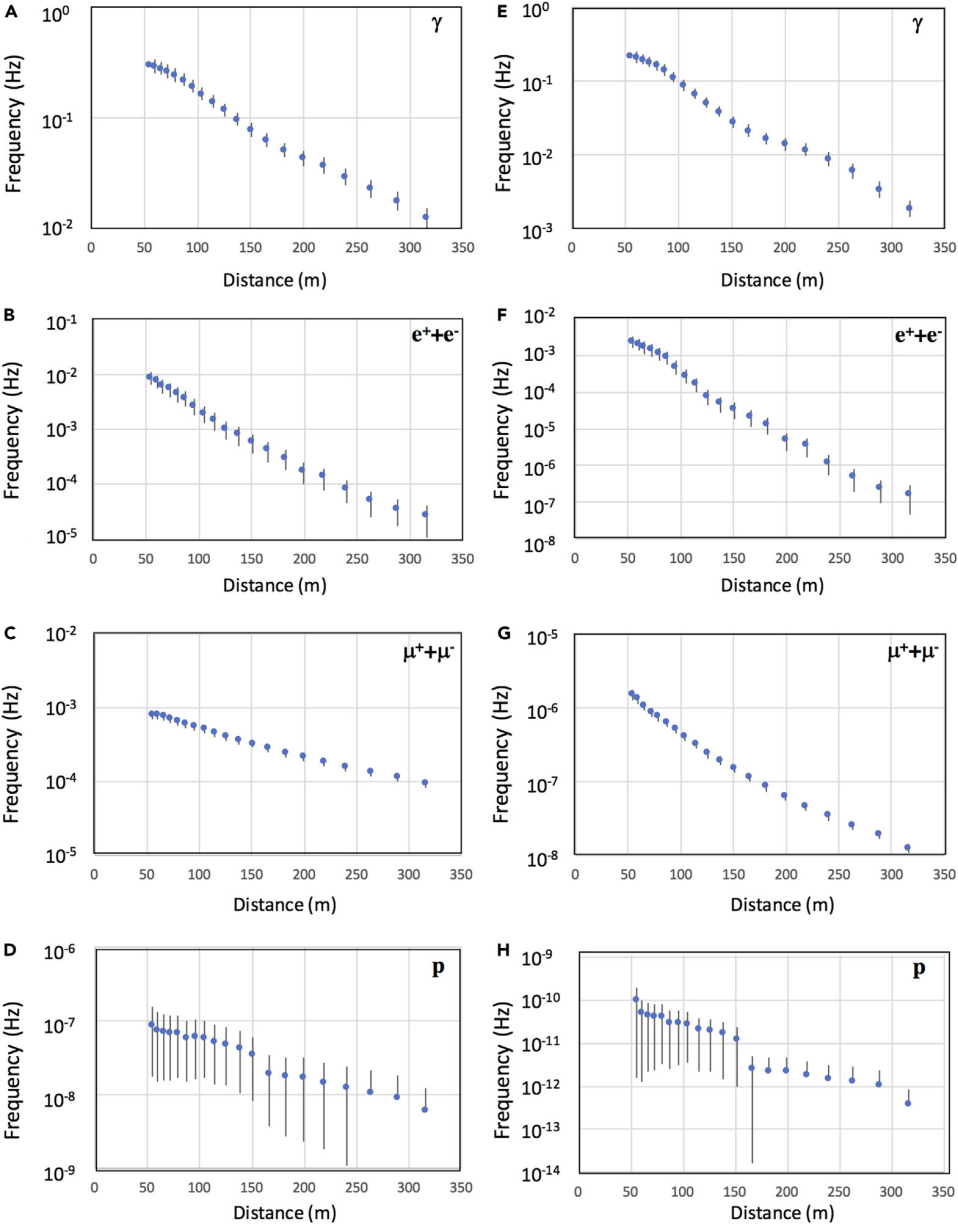
Monte Carlo simulation results for the dual coincidence rate and the triple coincidence rate as a function of the distant between detectors (A–H) The dual coincidence rates for gamma-rays (A), electrons/positrons (B), and muons (C), and protons (D) as well as the triple coincidence rates for gamma-rays (E), electrons/positrons (F), muons (G), and protons (H) are shown. Data points without negative error bars indicate that the lower limits of the error bars have reached zero.

The simulation procedure is summarized as follows:

(1) Considering the fact that the current size of our CTS network system coverage area is relatively small, 48,000 EASs were generated by injecting 48,000 primary particles (protons) with energies ranging from 10 TeV to 1 PeV into the simulated Earth’s atmosphere. The injection altitude was 100 km, and the ground level was set to be 1000 g cm^−2^. The showers initiated by either primaries lower than 10 TeV or primaries higher than 1 PeV were not considered because the number of EAS particles is not sufficient or the EAS frequency is too low for practical implementation of CTS in either case.
(2) The primary particles’ incident angle ranged from 0° to 90° from their zenith point, and the primary particles’ energy distribution was generated in accordance with the primary energy spectrum.^54^
(3) The cutoff energy for gammas, electrons/positrons, positive/negative muons, mesons, and nucleons were respectively set to be 80 keV, 80 keV, 10 MeV, 60 MeV, and 120 MeV. The areal densities of EAS particles were averaged over 48,000 EAS events.
(4) The dual coincidence rate and the triple coincidence rate between distant detectors were calculated by assuming 1-m^2^ detectors. The results for gamma rays and EAS charged particles (electrons/positrons, positive and negative muons, and protons) are shown in Figure 2. For a detector interval of 50 m, the dual coincidence rate and the triple coincidence rate were respectively 8.6 × 10^−3^ ± 2.3 × 10^−3^ Hz and 2.4 × 10^−3^ ± 8.0 × 10^−4^ Hz for EAS charged particles (except gamma rays). These coincidence events mostly come from electrons/positrons (99%), and other particle’s contributions are minor (muon; 1%, proton: 10^−3^%).

**Figure 3:**
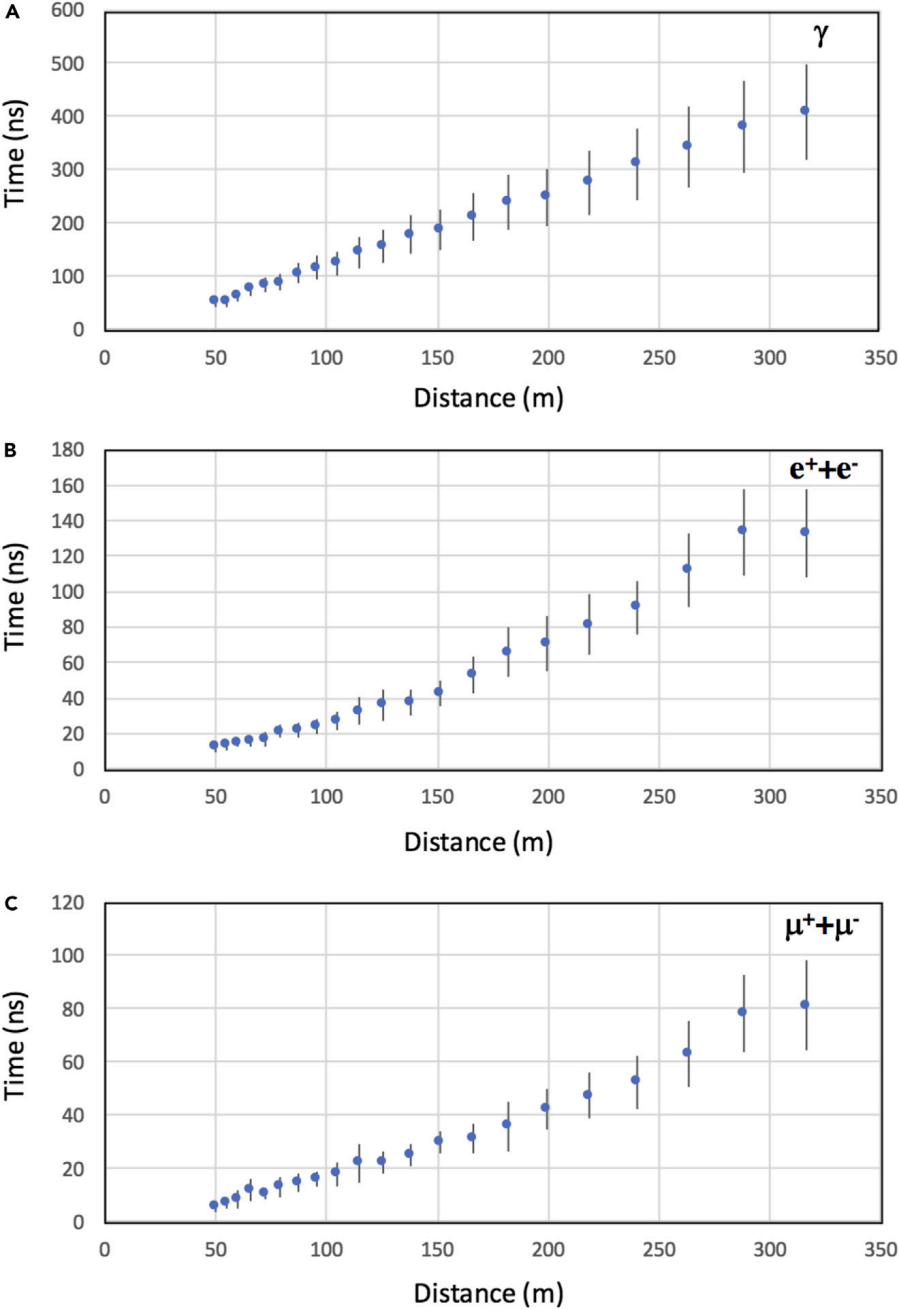
MonteCarlo simulation results of the EAS time structures (A–C) The time structures for gamma-ray (A), electron/positron (B), and muon showers (C) are shown. Blue circles and vertical bars respectively indicate the average delay of the particle arrivals and the disk thicknesses.

As is described later, the observed dual and triple coincidence rates were higher by a factor of ∼5 than these values estimated based only on EAS charged particles, probably because of a considerable number of γ-*e* conversions in the concrete slab located above the detectors. The thickness of the concrete slab located above the detector is roughly 2 radiation lengths. Therefore, it is expected that many of the gamma rays are converted to electron positron pairs before reaching the detector. Figure 3 shows the time structures (delay and disk thickness) for gamma-ray, electron/positron, and muon showers. If MCS and SCM are located on the circumference of the shower disk, there will be time delay between MCS and SCM; however, this plot was used for estimating the maximum values for time delay and associated fluctuations in arrival time. As can be seen in Figure 3, it was reasonable for us to assume that the time differences registered between CTS sensors for EAS events would be less than 100 ns.

### CTS principle of operation

After having reviewed the main aspects of EAS, in this section the principles of how CTS operates will be described. A remote CTS sensor with the local clocks (Ck2) to be compared with the standard clock (Ck1) detects EAS events at an absolute time (*t*_0_), but CTS sensors time stamp such events as *t*_ck1_ = *t*_0_ + δ*t*_1_ using Ck1 and as *t*_ck2_ = *t*_0_ + δ*t*_2_ + *r*_*t*2_ using Ck2. *t*_ck1_ and *t*_ck2_ differ from *t*_0_ because of the δ*t* component, coming from the EAS time structure (disk thicknesses and arriving directions) and furthermore, *t*_ck2_ further differs from *t*_0_ + δ*t*_2_ because of the local clock’s intrinsic time rate (*r*_*t*_), depending on their relative frequency offset and drift. The synchronization between the two clocks implies that *t*_ck1_ = *t*_ck2_ and, by transferring the time information *t*_ck1_ from ck1 to Ck2, Ck2 can be synchronized as:

(Equation 2) $$\left(t_{0}+{\delta t}_{2}+r_{t2}\right)\to \left(t_{0}+{\delta t}_{1}\right)\text{.}$$

As shown in this formula, because *t*_0_ is a common mode information the only component affecting the time synchronization is (*δt*_2_ - *δt*_1_).

Concerning (*δt*_2_ - *δt*_1_) estimation, the main aspects to be considered are:

(a) The EAS event rate that can be employed by CTS, depending on the relative frequency offset between the clocks to be compared and the detectors size.
(b) The lateral spread of the EAS.
(c) The CTS areal coverage.
(d) Fluctuations of the EAS arrival times.

### CTS sensors

**Figure 4:**
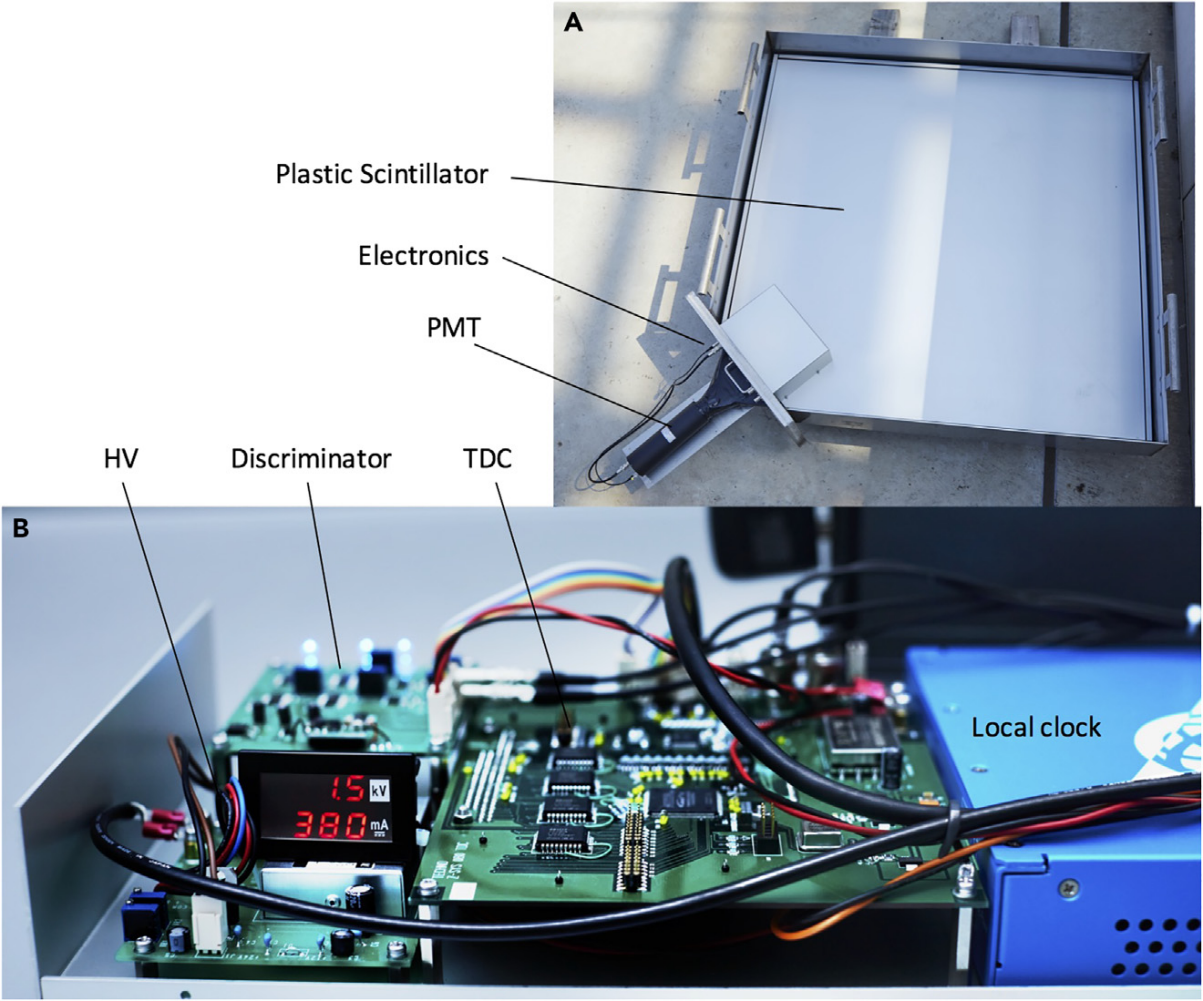
CTS sensor (A and B) The EAS detector and TDC modules are shown, respectively in (A and B).

CTS sensors have been devised, developed at tested at Tokyo University Laboratories. In the next Figures 4A and 4B, a picture of a CTS sensor considered in the present work is shown.

Dimensions and weight are (1,200 mm x 1,000 mm × 60 mm) and 20 kg, respectively. Each CTS sensor consists of an EAS detector and a TDC (Time to Digital Converter) module. The EAS detector is composed by a stainless-steel-made exterior case, a plastic scintillator sheet (1000 mm × 1000 mm x 20 mm), a 2-inch photomultiplier tube (PMT), an acrylic light guide and a lithium battery. The TDC module, on the other hand, consists of a TDC (ScioSence TDC-GPX), a signal scaler (Technoland Z-SYS), a high voltage power supply (HV), a discriminator, and a field programmable gate array (FPGA). In the current work the GPS OCXO Disciplined Oscillator (GPS DO | Trimble Thunderbolt PTP GM200) has been considered as the time and frequency reference.

When a charged particle hits a plastic scintillator sheet, the emitted scintillation photons propagate throughout the scintillator, being amplified by a PMT installed at the corner of the scintillator. PMT output signal is binarized by a discriminator and fed into the TDC STOP input, whereas the GPS DO 10 MHz output signal is fed into the TDC START input. In parallel to the TDC measurement, the total number (*n*) of 10 MHz OCXO cycles from the beginning of the measurements is counted by the signal scaler. When an event occurs, the discriminated PMT output is measured with respect to the GPS DO pulse, generating the Δ*T* measurement, with a jitter at the level of 1 ns. Once this measurement cycle is completed, the event can be time stamped as:

(Equation 3) $$t_{ck}=\Delta T+100\;ns\times n\text{,}$$

A time jitter because of the impact point of the particle in the scintillator is negligible (<10 ns), but the time fluctuations in discriminator’s outputs coming from variations in PMT pulse heights are much larger (∼50 ns S.D.) because we did not use constant fraction discriminators.

**Figure 5:**
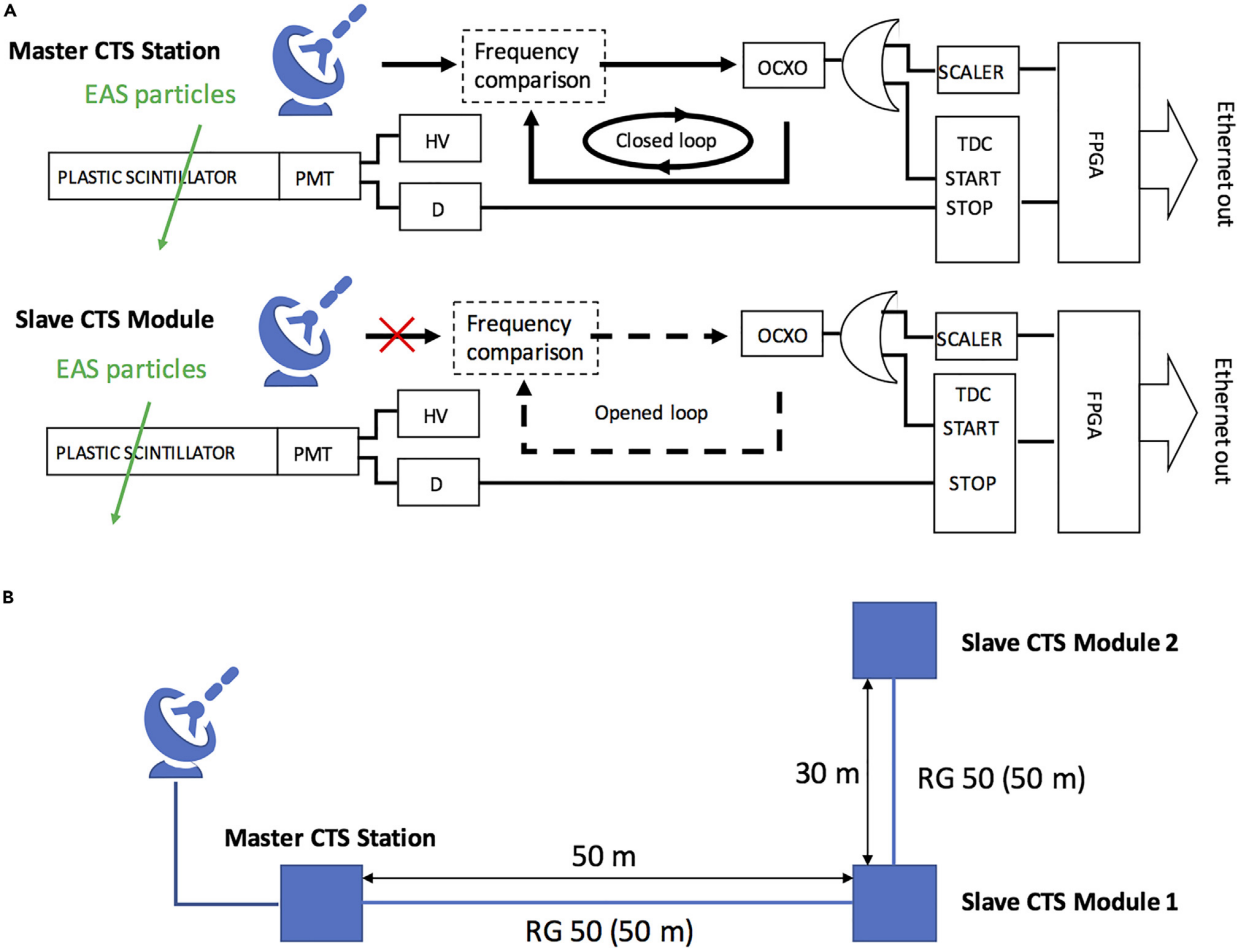
CTS sensors and the CTS Network System prototype (A) The antenna symbols indicate the GPS antenna. CLK1 and CLK2 are the local clocks associated with MCS, while CLK3 and CLK4 are the local clocks associated with SCM. A red cross mark indicates disconnection. (B) A geometrical configuration of the current CTS network system prototype is also shown. Blue filled squares and blue lines respectively indicate the CTS sensors and RG-50 coaxial cables connection.

In the following section Figure 5A, a functional scheme for the above-mentioned measurement process is shown.

### CTS network system

A CTS network system for time synchronization consists of at least two CTS sensors: one of which is designated as the MCS, because it is connected to a time reference, which could be either a standalone atomic clock, a GPS DO or a UTC(k) timescale; the other CTS sensors act as SCMs and are equipped with the local clocks to be synchronized. Each of the CTS sensors performs a local measurement, following the approach described in the previous section. Then, by comparing the MCS and SCMs timestamp data - called Event by Event Data (EED) - SCMs can find EAS coincidences and access to the MCS time reference - available in terms of timestamp data - every time an EAS coincidence is found. For this work, a prototype CTS network system based on three CTS sensors has been considered, deployed within 100 m intervals from each other at Tokyo University Laboratories. One acting as MCS, whereas the others as SCMs. In addition, all the CTS sensors have been connected by means of coaxial cables (RG-50, 50 m long) to compare a guaranteed synchronization results and CTS results. To go more into detail about the CTS sensors clocks, MCS was equipped with two GPS DOs that - by means of the TDC module - can be compared to each other and with respect to the EAS events. On the other hand, SCMs were equipped with GPS DOs, operating in free-running mode (they had been locked to GPS Time for evaluation purposes and - once - at the beginning of the measurement campaign).

The comparison between the two MSC GPS DOs was two-fold:

(a) Evaluation of the GPS Disciplined Oscillators (GPS DO) stability when locked to GPS Time.
(b) Evaluation of the GPS DO OCXO, by comparing one GPS DO locked to GPS Time, with respect to the other, operating in free running mode. After this evaluation, all the MCS GPS DOs have been operated locked to GPS Time.

Conversely, the comparison of MCS GPS DOs with respect to the EAS events by means of TDC is to transfer the GPS DOs time reference to the SCMs, allowing the required time synchronization.

### CTS data processing

**Figure 6:**
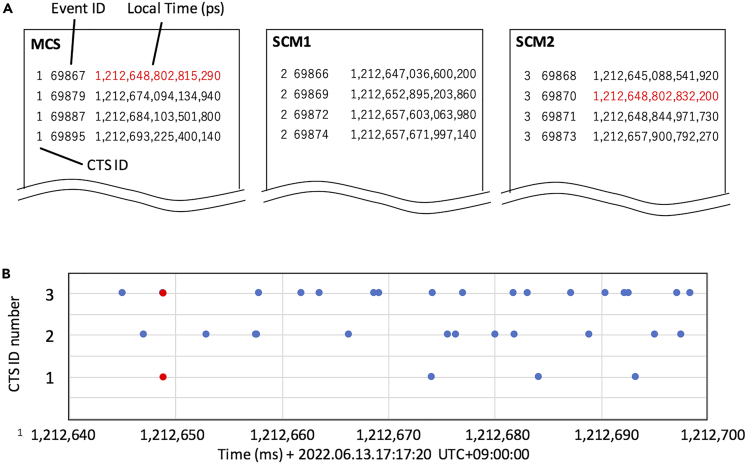
Example of the CTS sensor time stamp format (A) The red colored numbers show the local time that coincides within the time window of 1 μs. (B) The time sequential plots of the local EAS time at MCS, SCM1 and SCM2. Red filled circles indicate the local EAS time that coincides within the time window of 1 μs.

Figure 6A shows an example of the time stamp measurements provided by CTS sensors considered in the present work, acting as MCS, SCM1 and SCM2. Each time stamp record consists of the CTS sensor identification number, the event ID, and the time stamp information, corresponding to the EAS event arrival time (Δt + 100 ns x *n*). As can be seen in Figure 6A, most of the SCM time stamp information doesn’t match with the one provided by MCS. As mentioned in the previous sections, to perform required synchronization, MCS transfers its time stamp measurement to the SCMs, where a comparison process is carried out, aimed at finding pairs of coincidence measurements.

For a peer-to-peer data comparison, the procedure is the following:

(a) Set the time window (*T*_W_) to a large enough value to find coincidence events between SCM and MCS. A pair of corresponding measurements in MCS and SCM are called *t*_MCS_ and *t*_SCM_, respectively. It is important to note that *T*_W_ is determined by considering the local clocks relative frequency offset and the allowable accidental coincidence rate. As it will be described later, wider *T*_W_ values increase the accidental coincidence rate of single muons. Because the open-sky muon rate is ∼10^2^ Hz, arriving from the entire upper hemisphere per square meter, the accidental dual coincidence rate and accidental triple coincidence rate will be ∼2 × 10^4^ x *T*_W_ Hz and ∼3 × 10^6^ x *T*_W_^2^ Hz, respectively. On the other hand, if *T*_W_ is too narrow, the coincidence events will not be found when the time difference between the clocks at MCS and SCM exceeds *T*_W_.
(b) Correct *t*_SCM_, from *t*_SCM_ to *t*_MCS_. More specifically, the time stamp registered at MCS is replaced with the time stamp registered at SCM.
(c) Repeat the processes (a) and (b) every time coincidence events are found within the time window *T*_W_.

In order words, when an EAS coincidence is found, a synchronous event at MCS and SCMs is identified, allowing to adjust (swap) SCMs timestamps, to the MCS one, considered as the time reference. Of course, such time reference “transfer” from MCS to SCMs is affected by an uncertainty coming from the coincidences finding process, that depends mainly on the EAS time structure (i.e. (*δt*_2_ - *δt*_1_)).

For a peer-to-multiple-slave data comparison, the procedure is 2-fold:

(1) Finding multiple coincidence events instead of dual coincidence.
(2) Combining dual coincidence events with a daisy-chain configuration.

There are pros and cons for each of these methods. If a multiple coincidence procedure is applied, accidental coincidence events are drastically reduced. However, because the frequency at which multiple coincidence events are observed is much lower than the frequency at which dual coincidence events are observed, the resultant synchronization rate will be reduced. On the other hand, for 2), synchronization uncertainty is increased by a factor of a square root of the number of nodes between the uppermost stream SCM and the lower most stream SCM (*N*^1/2^).

Next, Figure 6B shows a time-sequential plot of the coincident events. In this figure, the events coinciding within *T*_W_ = 1.0 μs are indicated by red filled circles.

**Figure 7:**
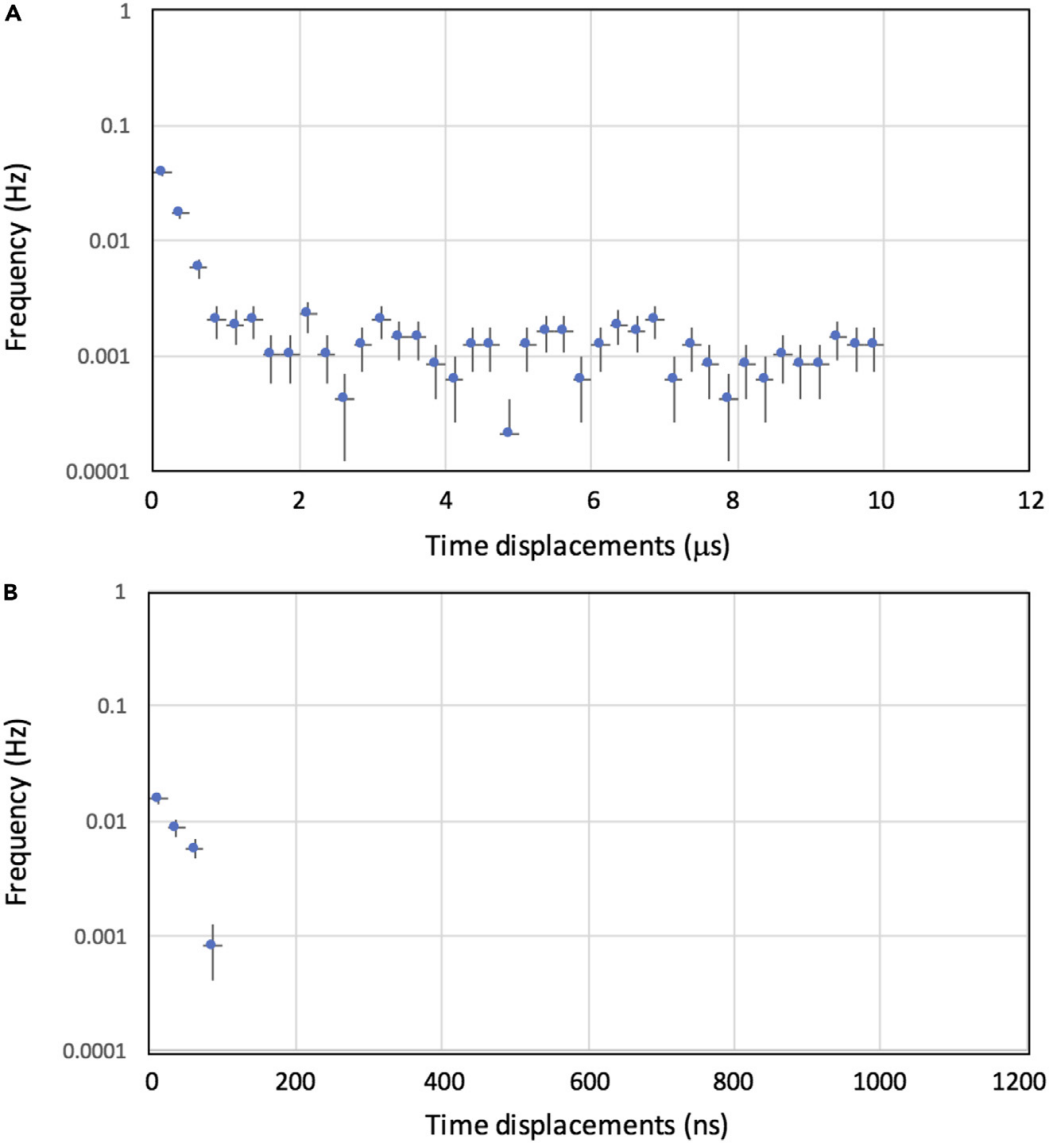
Distribution of the time differences These are the differences after selecting events by taking dual coincidence (A) and triple coincidence (B). The error bars associated with the data points are 1 Standard Deviation (SD).

Figure 7A shows the distribution of the time differences between SCM and MCS (|*t*_SCM_ - *t*_MCS_|) after selecting events by taking more than a two-fold coincidence with a time window *T*_W_ = 1.0 μs. The time required for transferring MCS EEDs and comparing them with the SCM ones is much shorter than the time difference between two showers, with a negligible effect on the CTS synchronization performances.

Figure 7B shows the distribution of the time differences between SCM and MCS (|*t*_SCM_ - *t*_MCS_|) after selecting events by taking three-fold coincidence, with a time window *T*_W_ = 1.0 μs. The triple coincidence rate was ∼30% of the entire coincidence rate. The constant background that can be seen beyond the range of 100 ns (∼0.04 Hz) comes from the accidental coincidences of open-sky events.

### CTS time synchronization results

**Figure 8:**
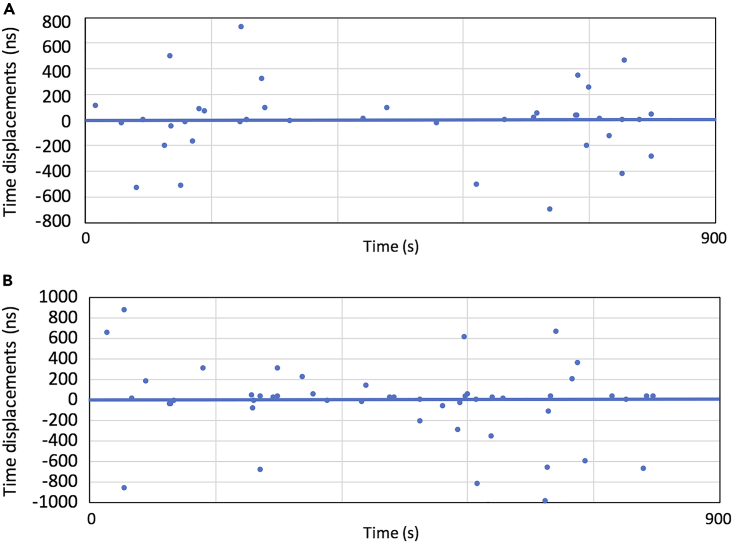
MCS time reference transfer to SCM, by means of dual-coincidence-based calibration factors (A and B) Calibration factors series generated through dual coincidences between MCS and SCM1 (A), and between MCS and SCM2 (B) are shown.

Starting from the considerations proposed above, the main aim of the current work was to conduct a preliminary evaluation of the time synchronization capabilities of the CTS technique, testing a short-haul prototype CTS network system, installed at the Tokyo University Laboratories. Figure 8 shows an example of MCS time reference as available at SCMs level every time an EAS event is detected, considering calibration factors series generated through dual coincidences between MCS and SCM1 (Figure 8A), and between MCS and SCM2 (Figure 8B). As already commented, the results of a numerical evaluation between the MCS time reference and the SCM’s time are affected by the (*δt*_2_ - *δt*_1_) uncertainty. Furthermore, as can be seen in Figure 8, although the rate of the dual coincidence events is high (0.04–0.07 Hz), the dual-coincidence-based calibration factors are not appropriate for practical use, because of a high accidental coincidence rate.

**Figure 9:**
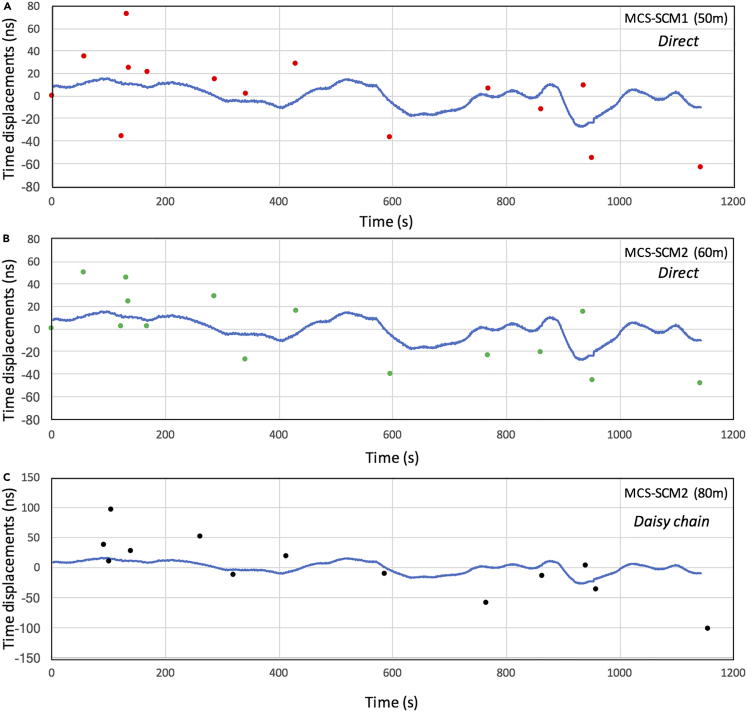
MCS time reference transfer to SCM, by means of triple-coincidence-based calibration factors The results are shown for MCS-SCM1 (50 m) (A), SCM2-MCS-SCM2 (60 m) (B) and SCM1-MCS-SCM2 (80 m) (adopting the MCS-SCMI1-SCM2 daisy-chain configuration) links (C).

Figure 9 shows MCS time reference as available at SCMs level every time an EAS event is detected (the colored dots), considering calibration factors determined by the triple coincidences between MCS, SCM1, and SCM2. For reference, the blue curve shows the actual MCS GPS DO behavior, with its typical relatively constant trend and about 20–30 ns peak-to-peak fluctuation. Calibration factors based on the MCS-SCM1-SCM2 daisy chain configuration have been also considered (Figure 9C).

As can be seen, although the rate of the triple coincidence events is reduced (∼0.02 Hz), better stability is achievable in the triple-coincidence-based calibration factors. In the next Table 1, Standard Deviation (SD) for MCS-SCM1 (50 m), MCS-SCM2 (60 m) and MCS-SCM2 (80 m) (adopting the MCS-SCM1-SCM2 daisy-chain configuration) links, is reported. As expected, an SD for this daisy-chain configuration link is degraded by a factor of 2^0.5^. In conclusion, for a daisy chain configuration (with an interval of 50–60 m) the synchronization performance between the uppermost node and the lowermost node is degraded as a function of the number of nodes. For instance, for a 1 km long daisy chain link with 20 nodes, a 138 ns- 157 ns calibration factors stability between the uppermost node and the lowermost node, must be expected. This is a natural consequence because the EAS arrival time is determined with a Poisonian distribution.

**Table 1:** CTS correction factors Standard Deviation (SD)

Time link	SD (1 σ)
MCS-SCM1 (50 m)	35 ns
MCS-SCM2 (60 m)	30.8 ns
MCS-SCM2 (80 m) (daisy-chain configuration)	48.3 ns

Considering that calibration factors allow to transfer MCS time reference to SCMs every time an EAS event is found, the SDs estimated above represent an estimator of the CTS synchronization capability in case the relative frequency offset between the SCMs clock (or any remote user clock to be synchronized) and MCS time reference, is reduced. In case the relative frequency offset is significant, CTS synchronization capabilities are deteriorated. Considering the typical rate of EAS events and the (*δt*_2_ - *δt*_1_) uncertainty magnitude, adopting a Rubidium as the oscillator for either the SCMs or the user, will most likely allow to keep CTS synchronization capabilities at the level of EAS characteristics (i.e. (*δt*_2_ - *δt*_1_) uncertainty), as per Figure 9.

In addition, SCMs clock (or any remote user oscillator) frequency steering to the MSC time reference, can be implemented. In this work, this feature has not been tested yet, but will be part of the next studies on CTS. Just as a hint, a possible approach to perform such a frequency steering could be the following one:

(1) Estimate relative frequency offsets averaged on EAS intervals. More specifically - if we define *T*_*i*_ as the interval between i^th^ EAS and (*i+1*)^th^ EAS, and D_i_ as the difference between the timestamp immediately before the (*i+1*)^th^ EAS and the i^th^ EAS correction factor - the relative frequency offset for the considered interval will be D_i_*T*_*i*_^−1^.
(2) Keep the relative frequency offset estimated from previous point until the next EAS event.
(3) When the next EAS event is recorded, repeat 1) and 2).

## Discussion

In this work, a preliminary evaluation of a short-haul CTS synchronization network system, installed at Tokyo University premises, has been carried out. Performances comparable to those obtainable with GPS DOs have been proved. MCS time reference (expressed in terms of timestamps), has been transferred to SCMs located 50–60 m away, at the level of ∼30 ns (SD, 1 σ), showing a behavior in line with expectations and preliminary simulations. Indeed, the observed dual and triple coincidence rates were much higher than those numerically estimated based only on EAS charged particles. This large gap between the numerical results and the experimental results can be explained by *γ*-*e* conversions initiated by the concrete slab located above the detectors. Because the current experiments were performed inside a building, thick concrete slabs above the detector must be considered. The thickness of the slab right above the detector is 30 cm, which is more than two times thicker than the radiation length (27 gcm^−2^) in SiO_2_; as such we expect that a substantial amount of electron positron pairs was generated right above the detector. The current results suggest that when the CTS network system is used in outdoor open-sky environments, the capability of time synchronization would be enhanced if a lead sheet or a steel sheet would be placed above the detector to promote electron positron pair production.

It was also found that although a peer-to-peer time synchronization based on dual coincidence events is an efficient process for CTS, because of the high frequency of dual coincidence occurrence, the rate of the accidental coincidence of open-sky muons increases, generating random timing information within the time range we set for coincidence (*T*_W_ = 1 μs). As can be seen in Figure 7, the accidental coincidence that generates erroneous timing information exceeding 500 ns is less than 20%, and this erroneous information would most likely be overwritten by the subsequent correct timing information within the next 20 s. However, adopting a dual coincidence approach would be impractical in many cases. In this work, this error rate was reduced by taking triple coincidence events. By doing so, these accidental coincidence intervals are reduced from ∼25 s to ∼3 days for the given coincidence time window (*T*_W_ = 1 μs). Considering the time rate of local clocks adopted in the present work, the coincidence time window could be reduced by 50%, leading to further reduction of the accidental coincidence rate. For a daisy chain configuration, the synchronization uncertainty is increased by a factor of *N*^0.5^, where *N* is the number of nodes.

**Figure 10:**
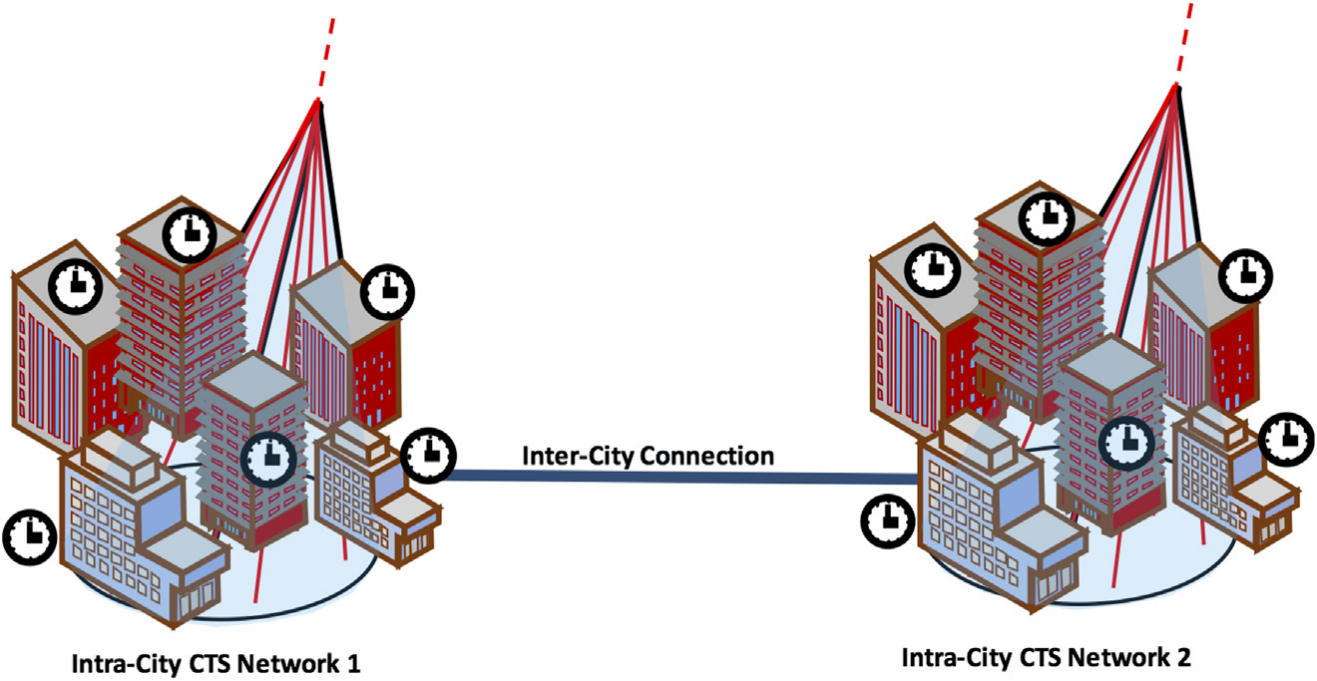
Conceptual view of a secured time synchronization scheme with intra-city CTS networks and inter-city wired connection

Because EAS particles arrive on a horizontal plane 2-dimensionally, it is more reasonable to design a 2-dimensional CTS network to synchronize local clocks within a city (or an area where it’s not convenient to deploy other time synchronization means), rather than a long distance (hundreds of miles) city-to-city connection with a 1-dimensional CTS daisy chain, because most of the EAS particles would be wasted in the latter case. Covering the area within the shower disk with a 2-dimensional CTS network is the most efficient way to maximize the potential of CTS. Therefore, creating a city-to-city CTS system is not recommended, but an intra-city CTS system would be cheaper than adopting optical-fiber-based systems. The costs required for establishing a new dedicated intra-city wired system for time synchronization, in fact, is high. For example, the costs per distance unit for the construction of a city new dedicated underground optical fiber network in Japan, would be around 2.8 MUSD/km for pipeline construction/civil engineering works and 0.7 MUSD/km for cable laying work (Organization for Cross-Regional Coordination of Transmission Operators, 2016). Therefore, establishing a wired optical fiber system, with 100 nodes at an interval of 60 m, would cost around 21 MUSD. On the other hand, establishing the same intra-city network adopting CTS technology - considering that each current CTS sensor costs around 6 k-15 k dollars - would require an economical effort less than 3–7% compared to the wired optical fiber system (600 k-1.5 M USD). Consequently, a combination of optical fiber wired, and CTS systems could be considered as a good tradeoff for granting secured time synchronization, for the users requiring timing performances in line with the ones provided by CTS. CTS clusters, in fact, could be established for intra-city synchronization, whereas (potentially already existing) dedicated optical fiber systems could be employed for the inter-city CTS connections and the dissemination of a reference timescale, like the ones generated at NMIs (Figure 10).

Hence, CTS could be configured to serve a potentially wide range of users and applications. In fact, CTS could be potentially conceived as a ‘self-adjusting’ technique offering high level continuous (perpetual) performances, to be considered either as a backup chain for GPS Disciplined Oscillators (GPS DO), a standalone reference standard for frequency and time interval measurements, or as a tool for the dissemination of reference time scales (e.g., physical realizations of UTC, namely UTC(k) to final users, with improved characteristics in terms of robustness and reliability.

In the latter case, the scenario would imply to have MCS connected to a time reference - like a UTC(k) - with SCMs installed at the user’s premises. CTS would be employed to disseminate time reference to the user, who would use the SCM clock 1PPS/10 MHz signals reproducing it. In this case, MCS could be hosted where the reference signals are generated (e.g., at NMIs) or at dedicated distribution nodes within the city/area to be served and reached by (already existing) optical fibers links. Best performances could be reached equipping SCMs, with Rubidium oscillators.

Another advantage of CTS is security. Because GPS signals are weak and unencrypted, they can easily be jammed and spoofed. Wired systems are much more robust, because they are located within intranets, possibly equipped with firewalls. However, unless these networks are totally independent from other network systems, there could be always the possibility to be hacked. On the other hand, CTS do not exchange actual time information and requires one set of two files like a key and a lock. The fact that the actual time information is computed at user level and measurements generated at different locations are required substantially increases the security. For example, to attempt to jam the CTS data, random data would have to be sent at a rate of more than 10 kHz, to significantly increase the accidental coincidence events that would mimic the real timing information. However, these files can be easily distinguished from the correct files that contain data at a rate of 100 Hz. In other words, it is impossible to find coincidence events between artificially generated data and the actual EAS data, if the data rate is ≲100 Hz. In Table 2, pros and cons of CTS and other time synchronization technologies are compared.

**Table 2:** Comparison between CTS and other time synchronization techniques

Technique	Accuracy	Security	Cost
CTS (current work)	∼30 ns	High	Low
GPS-DO (current work)	∼10 ns	Low	Low
Atomic Clocks (Cs)^a^	3 μs yr^−1^	High	Middle
WLAN^b^	200 ns-76 μs	Middle	Low
Optical Fibers^c^	100 ps km^−1^ K^−1^	High	High

a Zhan et al.^55^

b Cena et al.^56^; Chang et al.^57^; Yang et al.^58^; Carli & Zampieri^59^; Masood et al.^60^

c Serizawa et al.^16^

With this work, we preliminary investigated the time synchronization performances achievable with CTS, on real measurements generated by a short-haul prototype CTS network system installed at Tokyo University. CTS can be considered as a robust tool for time synchronization and dissemination applications, with performances comparable with the ones achievable by means of GPS DOs. Further works will test the CTS network system capabilities by connecting MCS to better time references (e.g., UTC(k)), considering different types of SCMs local clocks (ranging from OCXO, to Rubidium, up to Active Hydrogen Masers and – possibly - UTC(k) time scales), as well as increasing the distance among the sensors and conceiving underwater installations. The feature to perform the physical steering of the SCMs local clocks will be investigated, as well the possibility to adopt new types of EAS detectors for CTS sensors, in the perspective to make CTS even more attractive in terms of size and costs, also increasing the possibility to make it a very capillary system, with a moderate economic effort. Finally, the possibility to test and meteorologically characterize CTS at NMIs, will be also envisaged.

### Limitations of the study

The nodal distance between SCM and MCS is limited to 60 m in this study. The total distance between SCM and MCS is limited to 80 m in this study. Time synchronization accuracy is limited to ∼30 ns (SD) in this study.

## STAR★Methods

### Key resources table

**Table:** 

REAGENT or RESOURCE	SOURCE	IDENTIFIER
**Other**		

PMT	Hamamatsu Photonics	R7724
Plastic scintillator	Eljen Technology	EJ-200
TDC	ScioSense	GPX
GPS-DO	Trimble	Thunderbolt GM200

### Resource availability

#### Lead contact

Further information and requests for resources and reagents should be directed to and will be fulfilled by the lead contact, Hiroyuki K.M. Tanaka (ht@eri.u-tokyo.ac.jp).

#### Materials availability

This study did not generate new unique reagents.

### Experimental model and subject details

Our study does not use experimental models typical in the life sciences.

### Method details

#### Material preparation

Our study does not materials typical in the life sciences.

#### Experimental apparatus

See cts sensors and cts network system sections.

#### Experimental procedure

See cts data processing section.

### Quantification and statistical analysis

There is no statistical analysis in this paper.

### Additional resources

We have no relevant resources.

## Data Availability

The datasets used and/or analyzed during the current study available from the corresponding author on reasonable request.
